# Augmenter of liver regeneration protects the kidney from ischaemia‐reperfusion injury in ferroptosis

**DOI:** 10.1111/jcmm.14302

**Published:** 2019-04-16

**Authors:** Li‐li Huang, Xiao‐hui Liao, Hang Sun, Xiao Jiang, Qi Liu, Ling Zhang

**Affiliations:** ^1^ Department of Nephrology The Second Affiliated Hospital Chongqing Medical University Chongqing People's Republic of China; ^2^ Key Laboratory of Molecular Biology for Infectious Diseases (Ministry of Education) Department of Infectious Diseases The Second Affiliated Hospital Institute for Viral Hepatitis Chongqing Medical University Chongqing People's Republic of China

**Keywords:** acute kidney injury (AKI), augmenter of liver regeneration (ALR), ferroptosis, ischaemia‐reperfusion (I/R) injury, reactive oxygen species (ROS)

## Abstract

Acute kidney injury (AKI) is a common and severe clinical condition with high morbidity and mortality. Ischaemia‐reperfusion (I/R) injury remains the major cause of AKI in the clinic. Ferroptosis is a recently discovered form of programmed cell death (PCD) that is characterized by iron‐dependent accumulation of reactive oxygen species (ROS). Compelling evidence has shown that renal tubular cell death involves ferroptosis, although the underlying mechanisms remain unclear. Augmenter of liver regeneration (ALR) is a widely distributed multifunctional protein that is expressed in many tissues. Our previous study demonstrated that ALR possesses an anti‐oxidant function. However, the modulatory mechanism of ALR remains unclear and warrants further investigation. Here, to elucidate the role of ALR in ferroptosis, ALR expression was inhibited using short hairpin RNA lentivirals (shRNA) in vitro model of I/R‐induced AKI. The results suggest that the level of ferroptosis is increased, particularly in the shRNA/ALR group, accompanied by increased ROS and mitochondrial damage. Furthermore, inhibition of system xc‐ with erastin aggravates ferroptosis, particularly silencing of the expression of ALR. Unexpectedly, we demonstrate a novel signalling pathway of ferroptosis. In summary, we show for the first time that silencing ALR aggravates ferroptosis in an in vitro model of I/R. Notably, we show that I/R induced kidney ferroptosis is mediated by ALR, which is linked to the glutathione‐glutathione peroxidase (GSH‐GPx) system.

## 1 INTRODUCTION

Acute kidney injury (AKI) is a common and severe clinical condition with high morbidity and mortality worldwide.^1^, ^2^ Moreover, AKI from ischaemia‐reperfusion (I/R) injury is the main cause of AKI under common clinical conditions.^3^, ^4^, ^5^ The pathogenesis of AKI is multiphasic and multifactorial, including renal endothelial injury, microvascular dysfunction,^4^, ^6^ and oxidative stress, all of which contribute to renal tubular epithelial cell (RTEC) death.^7^, ^8^ All previous studies have mainly been based on autophagy, apoptosis, or necrosis. However, recently, increasing evidence suggests that ferroptosis plays an important role in RTEC injury.^9^, ^10^, ^11^, ^12^, ^13^, ^14^ It has been demonstrated that the inactivation of glutathione peroxidase‐4 (GPX_4_), a ferroptosis‐associated gene, triggers AKI in mice.^15^ Additionally, the regulator GPX_4_ is necessary to prevent renal tubular cell injury in ferroptosis.^9^ Renal tubular cell of ferroptosis could be inhibited in vivo by third‐generation ferrostatin (SRS 16‐86), a potent antioxidant molecule.^10^ Moreover, ferroptosis was the main cause of folic acid‐induced AKI in mice, not necroptosis.^12^ Taken together, ferroptosis plays a critical role in AKI.

Ferroptosis is a newly discovered type of regulated cell death (RCD), which is characterized as an iron‐independent form of cell death that is morphologically, genetically, and biochemically distinct from necrosis and apoptosis.^16^ Ferroptosis is triggered by inactivation of cellular glutathione (GSH)‐dependent antioxidant defences, leading to the accumulation of lipid peroxidative products and toxic levels of reactive oxygen species (ROS).^17^, ^18^, ^19^ Several regulators of ferroptosis have been identified in some cells. Notably, GPX_4_, which is unique among GPXs isoforms, is the only enzyme that regulates the lipid ROS balance during ferroptosis.^20^ Additionally, GPX_4_ enzymatic activity depends on the cystine/glutamate antiporter system xc‐.^21^ In summary, ferroptosis is involved in AKI, and suppression of ferroptosis may provide new therapeutic strategies for AKI. However, this study examines the mechanisms underlying ferroptosis in AKI.

Augmenter of liver regeneration (ALR) is a widely distributed multifunctional growth factor that is expressed in all mammalian tissues, including the kidney.^22^, ^23^, ^24^ Originally, ALR was purified from the liver of new‐born rats, and a major function of this protein is to promote hepatocyte proliferation and liver regeneration.^25^, ^26^ Increasing evidence has shown that ALR plays a protective role against apoptosis and oxidative effects by inhibiting cytochrome *c* mitochondrial release and enhancing Adenosine Triphosphate (ATP) levels.^25^, ^27^, ^28^ We recently demonstrated that ALR expression was significantly increased after ischaemic and/or gentamicin toxicity in the renal cortex of rats with AKI.^22^, ^29^ Moreover, an intraperitoneal injection of recombinant human ALR (rhALR) enhanced the proliferation of renal tubular cells and significantly attenuated tubular cell apoptosis.^30^ Our previous studies indicated that ALR played a protective role by reducing the generation of ROS. Notably, the accumulation of ROS is the key trigger of ferroptosis, although the mechanism by which ROS accumulation elicits cell ferroptosis is not clear. Mechanistically, an intriguing issue that remains to be resolved is whether or how ALR displays protective activity against ferroptosis in I/R kidney injury. Regrettably, no studies to date have documented the effects of ALR on ferroptosis in AKI. In our present study, ALR was knocked‐down using short hairpin RNA (shRNA) lentiviral vectors to investigate the role of ALR in ferroptosis in human proximal tubular cells.

## 2 MATERIALS AND METHODS

### 2.1 Cell culture and mode of H/R in vitro

Human kidney proximal tubular (HK‐2) cells were purchased from the American type culture collection (ATCC, Rockville, USA) and cultured in Dulbecco's minimum essential medium plus F12 (DMEM/F12, BI, Israel) supplemented with 10% foetal bovine serum (FBS; Gibco, Carlsbad, CA, USA) and 1% penicillin/streptomycin (Invitrogen, Carlsbad, CA, USA) in an incubator under an atmosphere of 5% CO_2_ at 37°C. To induce H/R injury in vitro, cells were synchronized for growth in serum‐free media overnight and then maintained in serum‐free and D‐glucose‐free media in a hypoxia chamber (Thermo Fisher, MA, USA) with 94%N_2_, 5%CO_2_ and 1% O_2_ for 6 hours. After 6 hours, HK‐2 cells were moved to the standard chamber and supplied with complete media at 37°C for the indicated times (3, 6, 12 and 24 hours). If necessary, erastin (5 μmol/L) was administered 12 hours before H/R treatment. At the end of the treatment, cells were collected at the indicated time‐points for biochemical analyses.

### 2.2 shRNA lentiviral infection

The ALR‐targeted shRNA lentivirus with puromycin (LV‐ALR shRNA; GFER‐41526‐1) and control shRNA lentivirus (LV‐shRNA; CON054) were obtained from GeneChem (Shanghai, China). HK‐2 cells were plated in 6 cm culture dishes and then transfected with shRNA/ALR or shRNA/control at a multiplicity of infection (MOI) of 6 for 72 hours according to standard protocols. After 3 days of transduction, successfully transduced cells were selected. For stable transduction, transduced cells were cultured in 3 μg/mL puromycin (Sigma‐Aldrich Corporation) for the next experiment. Western blotting and real‐time quantitative polymerase chain reaction (PCR) were performed to verify the efficiency of infection.

### 2.3 Transmission electron microscopy (TEM) examination

HK‐2 cells were harvested immediately after the indicated treatment. HK‐2 cells on a 10 cm dish were fixed in 2.5% glutaraldehyde for 1 hour at room temperature, post‐fixed with 1% OsO_4_, dehydrated through a graded series of ethanol solution for 15 minutes and then embedded in epoxy resin, sliced into 60 nm continuous sections, stained with 2% uranyl acetate and lead citrate, and observed using TEM (Hitachi, Tokyo, Japan).

### 2.4 Evaluation of cell viability

Cell growth and viability were detected with a cell counting kit (CCK‐8, dojindo). According to the manufacturer's recommendations, normal and infected lentiviral cells were plated at a density of 1 × 10^4^ per well in 96‐well plates and observed at 0, 24, 48 and 72 hours. The optical density (OD) at a wavelength of 450 nm was measured using a microplate reader (Molecular Devices, LLC, Sunnyvale, CA, USA). The percentage of living cells was calculated according to the ratio of the absorbance of the experimental group to that of the normal group. The experiment was performed three times under the same conditions.

### 2.5 Western Blot

Cells were washed with PBS, and total protein lysates were collected using a total protein extraction kit and then quantified with the BCA protein assay reagent (KenGen Biotech Co. Ltd., Nanjing, China). Total protein samples (20 μg/lane) were separated by 10%‐15% SDS‐PAGE (Bio‐Rad, USA) and transferred onto a polyvinylidene difluoride (pore size 0.25 μmol/L) membrane at a constant current of 260 mA. After blocking with 5% bovine serum albumin (BSA) for 1 hour, the membranes were incubated with specific primary antibody transferrin receptor (TFR; ab214039, Abcam; ACSL4, ab155282, Abcam; xCT, ab175186, Abcam; GPX4, ab125066, Abcam; ALR, PA5‐48256, Thermo Fisher) at 4°C overnight or 2 hours at room temperature. After washing with Tris‐buffered saline Tween 20 (TBST) three times, all membranes were incubated with peroxidase‐conjugated secondary antibodies for 1 hour at room temperature. Finally, the membranes were washed three times and visualized using the Chemi Doc Imaging System (Bio‐Rad, USA).

### 2.6 Quantification of mRNA expression by real‐time PCR

Cells were purified using Total RNA extraction kits (Sigma‐Aldrich Corporation) according to the manufacturer's instructions. The concentration of RNA was measured and then reversed transcribed with a reverse transcription kit (Takara, Japan). The sequences of the gene‐specific primers were as follows: ALR: 5′‐GTGAGGAGTGTGCTGAAGACCT‐3′ and 5′‐TGAGCAGTCGAAGTCAGGCTTG‐3′ and GAPDH: 5′‐TGACTTCAACAGCGACACCCA‐3′ and 5′‐CACCCTGTTGCTGTAGCCAAA‐3′. To quantify gene expression, a relative quantification method, 2^−∆∆Ct^, was used.

### 2.7 Co‐immunoprecipitation

Total proteins extracted from HK‐2 cells were lysed in lysis buffer and quantified using the Coomassie plus protein assay reagent, and aliquots were stored at −80°C. Briefly, immunoprecipitation was performed with total protein lysates (600 μg) and the appropriate antibody overnight at 4°C on a rotator. The antibodies were as follows: GPX_4_ monoclonal antibody (1:100, Thermo Fisher), ALR polyclonal antibody (1:50, Santa Cruz biotechnology) and IgG (1:50, CST). The next day, the agarose beads were washed three times in buffer (50 mmol/L Tris (pH7.4), 150 mmol/L NaCl, 1% Nonidet P‐40 and 0.1% SDS) and two times in TBS, and then, 100 μL of a 20% slurry of Sepharose beads (Abcam, UK) in lysis buffer was added to the mixture and rocked for 2 hours at 4°C on a rotator. Then, the protein complexes bound to Sepharose beads were centrifuged at 2504 *g* for 5 minutes at 4°C. Finally, the bound proteins were mixed with an equal volume of 1 × buffer at 95°C for 5 minutes. An aliquot (30 μL) of the supernatant was fractionated by SDS‐PAGE using 15% acrylamide gels and Coomassie stained or transferred to polyvinylidene fluoride (PVDF) and blotted with the indicated antibodies.

### 2.8 Cell immunofluorescence

To observe GPX_4_ and ALR colocalization, immunofluorescence analysis was performed with an anti‐ALR monoclonal antibody (Santa Cruz Biotechnology) and anti‐GPX_4_ polyclonal antibody (Thermo Fisher). HK‐2 cells were seeded on a confocal dish. In addition, mitochondria were visualized using the mitochondria‐specific fluorescent dye TMRM (100 μmol/L). Cells were stained with TMRM for 20 minutes before fixation and then permeabilized with 0.3% (v/v) Triton X‐100 for 10 minutes. After three washes with PBS, cells were blocked with 5% BSA and incubated with different primary antibodies (GPX_4_, 1:200 in PBS, ALR, 1:50 in PBS) overnight at 4°C overnight with rocking. The cells were washed with PBS and then incubated with FITC‐labelled anti‐rabbit IgG (1:50) and TRITC‐labelled antimouse IgG (1:50) for 1 hour in the dark. Then, cell nuclei were counterstained with 4,6‐diamidino‐2‐phenylindole (DAPI, 300 nmol/L) for 10 minutes. The cells were washed with PBS, and a drop of Prolong Gold anti‐fade reagent was added before visualization using a Nikon laser scanning confocal microscopy. For each experimental setting, immunofluorescent images were captured with identical light exposure times. All colocalization studies were blinded.

### 2.9 Detection of cellular ROS

The intracellular ROS level was measured using a total reactive oxygen species (ROS) assay kit. After treatment with or without H/R or Fer‐1 (Sigma‐Aldrich), cells were exposed to 20 μmol/L 2′7′‐dichlorofluorescin diacetate (DCFH‐DA, Sigma, USA), incubated in an incubator for 20 minutes with gentle shaking every 5 minutes, and washed three times with serum‐free media. All cells were collected by centrifuging at 626 *g* for 5 minutes and one wash with PBS. The fluorescence intensity was determined by flow cytometry at an excitation wavelength of 488 nm and emission wavelength of 525 nm. The intracellular ROS levels in each group were presented as the mean fluorescence intensity. Three independent trials were performed.

### 2.10 Glutathione peroxidases (GPXs) determined

Cells in a 60 mm dish with or without H/R treatment were washed twice with PBS. The cells were then detached with a cell scraper in PBS, centrifuged at 626 *g* for 5 minutes and resuspended in 100 μL of cell lysis solution and homogenized in lysis buffer on ice for 15 minutes. Crude lysates were centrifuged at 12 000 g for 10 minutes to obtain the supernatant to measure the total amount of protein by BCA assay. Part of the supernatant sample was used to measure the activity of glutathione peroxidase with a total glutathione peroxidase assay kit (Beyotime, China). Briefly, glutathione reductase, GPXs assay buffer, sample and peroxide reagent tertbutylhydroperoxide (t‐Bu‐OOH) were mixed together and measured at 340 nm at room temperature.

### 2.11 Statistical analysis

All quantitative data are expressed as the means ± SD using GraphPad Prism 5.0 software (GraphPad Software, Inc., San Diego, CA, USA) for our study. One‐factor analysis of variance (ANOVA) followed by Tukey's post hoc test was used to analyse the variance for multiple group comparisons. Single asterisk and double asterisk indicate statistically significant at *P *<* *0.05 and *P *<* *0.01, respectively.

## 3 RESULTS

### 3.1 I/R induced ferroptosis and ALR was up‐regulated

**Figure 1:**
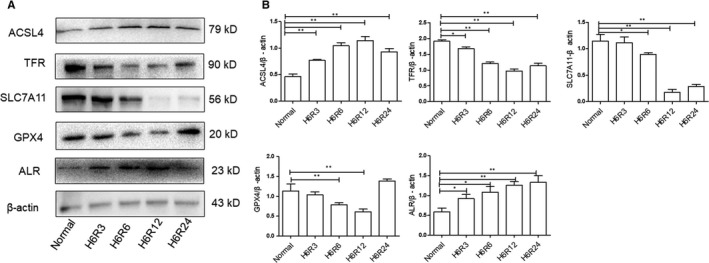
Ferroptosis was more apparent at H6R12 h, and augmenter of liver regeneration (ALR) was up‐regulated after I/R injury. A, Representative Western blot demonstrating the expression of acyl‐CoA synthetase long‐chain family member 4 (ACSL4), transferrin receptor (TFR), SLC7A11, glutathione peroxidase (GPX)_4_ and ALR. B, The ratio of ACSL4, TFR, SLC7A11, GPX_4_ and ALR was quantified by densitometry based on immunoblot images and depicted as the radio of the indicated proteins normalized to β‐actin. *P < 0.05 and **P < 0.01 compared to the normal group

Ferroptosis is a metabolic disorder of lipid peroxidation; therefore, lipid peroxides cannot undergo GSH metabolism catalysed by GPX_4_, which produces a large amount of toxic lipid ROS.^18^, ^31^ To explore the level of ferroptosis and ALR expression in HK‐2 cells after I/R treatment, the expression of acyl‐CoA synthetase long‐chain family member 4 (ACSL4), transferrin receptor (TFR), SLC7A11, GPX_4_ and ALR was determined by Western blotting (Figure 1A). The results showed that the ALR protein was significantly up‐regulated after I/R injury, peaking at 12 hours. Additionally, similar results were obtained by real‐time PCR analysis of HK‐2 cells (Figures 1B). Taken together, the results indicated that renal ischaemia‐reperfusion injury included ferroptosis.

### 3.2 Changes of the ROS levels and Fer‐1 attenuate the ROS levels after I/R

**Figure 2:**
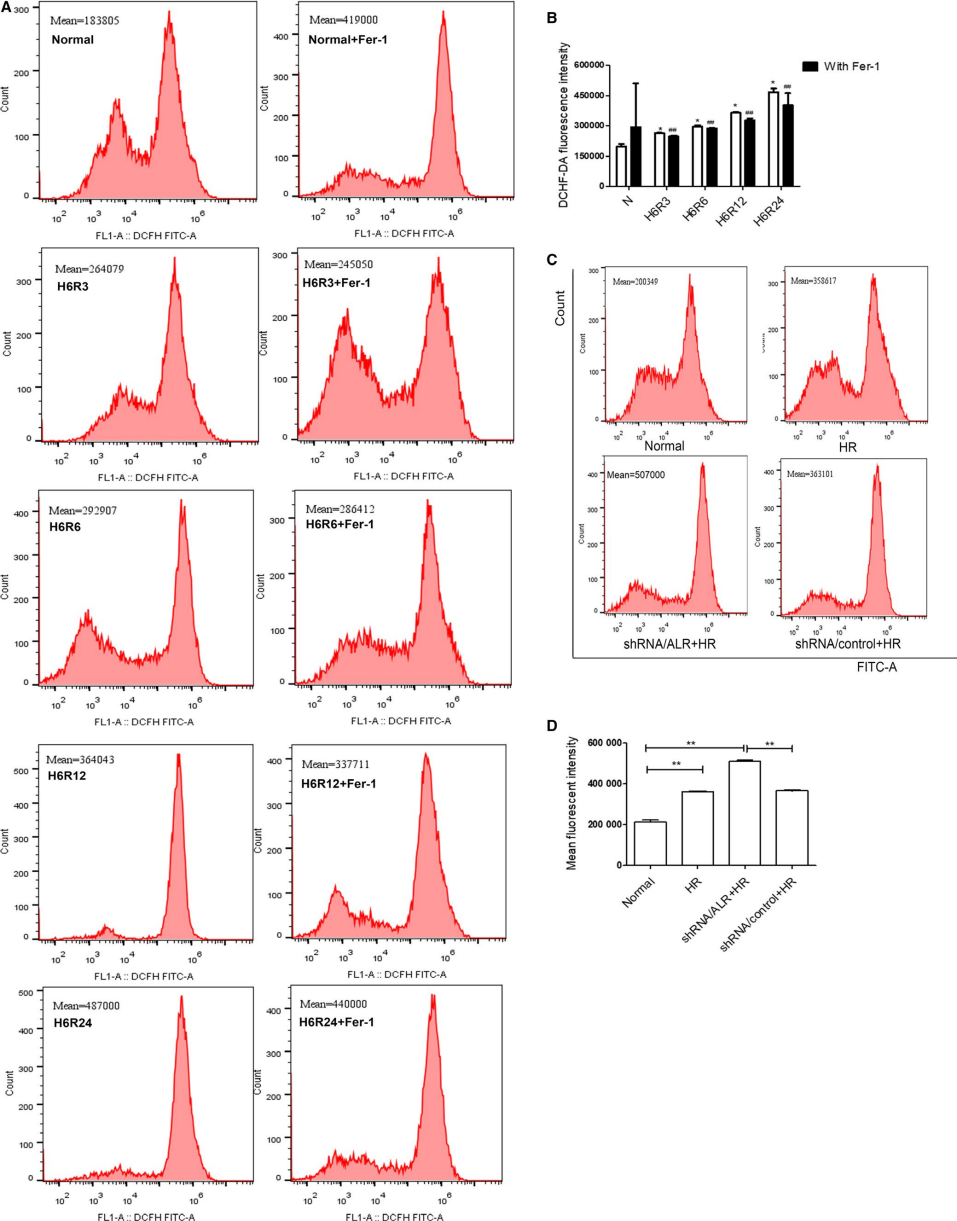
Reactive oxygen species (ROS) levels in Human kidney proximal tubular cells were evaluated using a dichloro‐dihydro‐fluorescein diacetate (DCFH‐DA) kit. A, C, ROS levels were measured by flow cytometry. B, D, The mean ROS fluorescence intensities in the short hairpin RNA lentivirals (shRNA)/augmenter of liver regeneration and shRNA/control groups in vitro. The mean ± SD represents three independent experiments (n = 3). *P < 0.05 and **P < 0.01 compared to the normal group. ^##^ *P* < 0.05 as compared to the hypoxia‐reoxygenation group at the same time‐point

We hypothesized that ferroptosis is a novel type of ROS‐associated cell death, and our previous studies showed that ALR could enhance ROS scavenging and eliminate oxidative stress. Therefore, the intracellular ROS levels in HK‐2 cells were detected, which showed that intracellular ROS generation was significantly increased in HK‐2 cells treated for longer times. Moreover, the ROS levels were attenuated by Fer‐1 (0.5 μmol/L; Figure 2). Additionally, silencing ALR in HK‐2 cells caused a significant increase in the intracellular ROS levels under the H/R condition compared with those of the shRNA/control group (*P <* *0.05).

### 3.3 Silencing of ALR in HK‐2 cells

**Figure 3:**
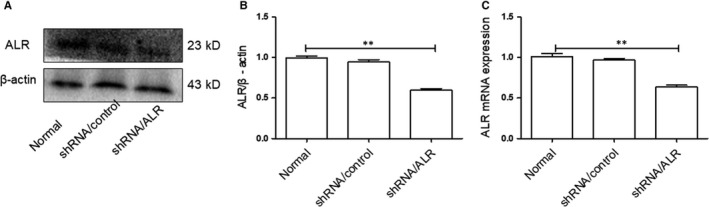
Effect of short hairpin RNA lentivirals (shRNA)/augmenter of liver regeneration (ALR) on ALR expression. Human kidney proximal tubular cells were transduced with shRNA/ALR or shRNA/control for 72 h, puromycin selected, and tested for ALR expression. A, B, Representative Western blot demonstrating the expression of ALR normalized to β‐actin. C, To evaluate the knockdown of ALR mRNA expression, real‐time PCR was performed and normalized to β‐actin. **P < 0.01 versus the normal group

Lentivirus transduction of HK‐2 with ALR shRNA/ALR leads to specific and stable down‐regulation of ALR expression. ALR expression was evaluated by Western blotting (Figure 3A) and real‐time PCR (Figure 3C). Western blot analysis showed that ALR was significantly down‐regulated in the shRNA/ALR group compared with those in the normal group. Similar results were obtained by real‐time PCR.

### 3.4 Silencing of ALR did not affect cell viability

**Figure 4:**
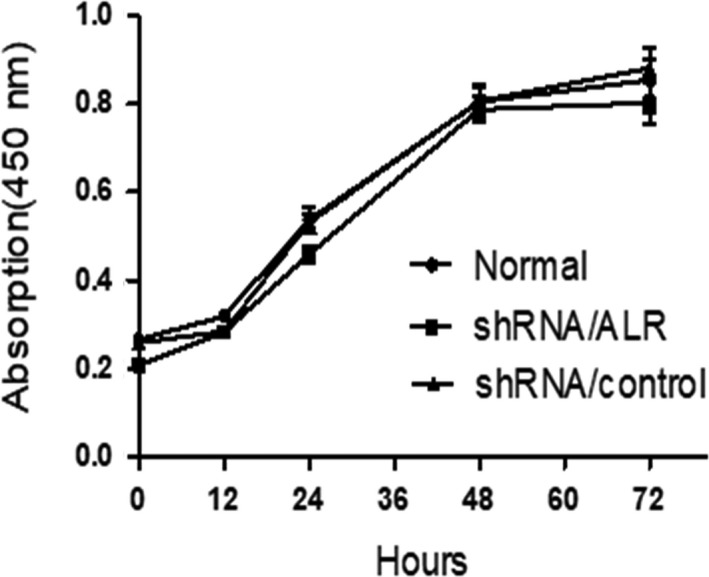
Cell counting kit (CCK8) assay of cell proliferation. Human kidney proximal tubular (HK‐2) cells transfected with short hairpin RNA lentivirals (shRNA)/control or shRNA/augmenter of liver regeneration (ALR) for the indicated times and analysed by using colorimetry at 450 nm. No significant difference in HK‐2 proliferation was observed between the shRNA/ALR group and shRNA/control group

To determine whether shRNA/ALR affected cell proliferation, a CCK‐8 assay was performed at different time‐points. The results showed that knockdown of ALR expression did not affect HK‐2 cell proliferation, with no significant differences in cell viability between the shRNA/control group and the normal group at 0, 24, 48 and 72 hours (*P *> 0.05, Figure 4).

### 3.5 Silencing of ALR up‐regulated ferroptosis in vitro

**Figure 5:**
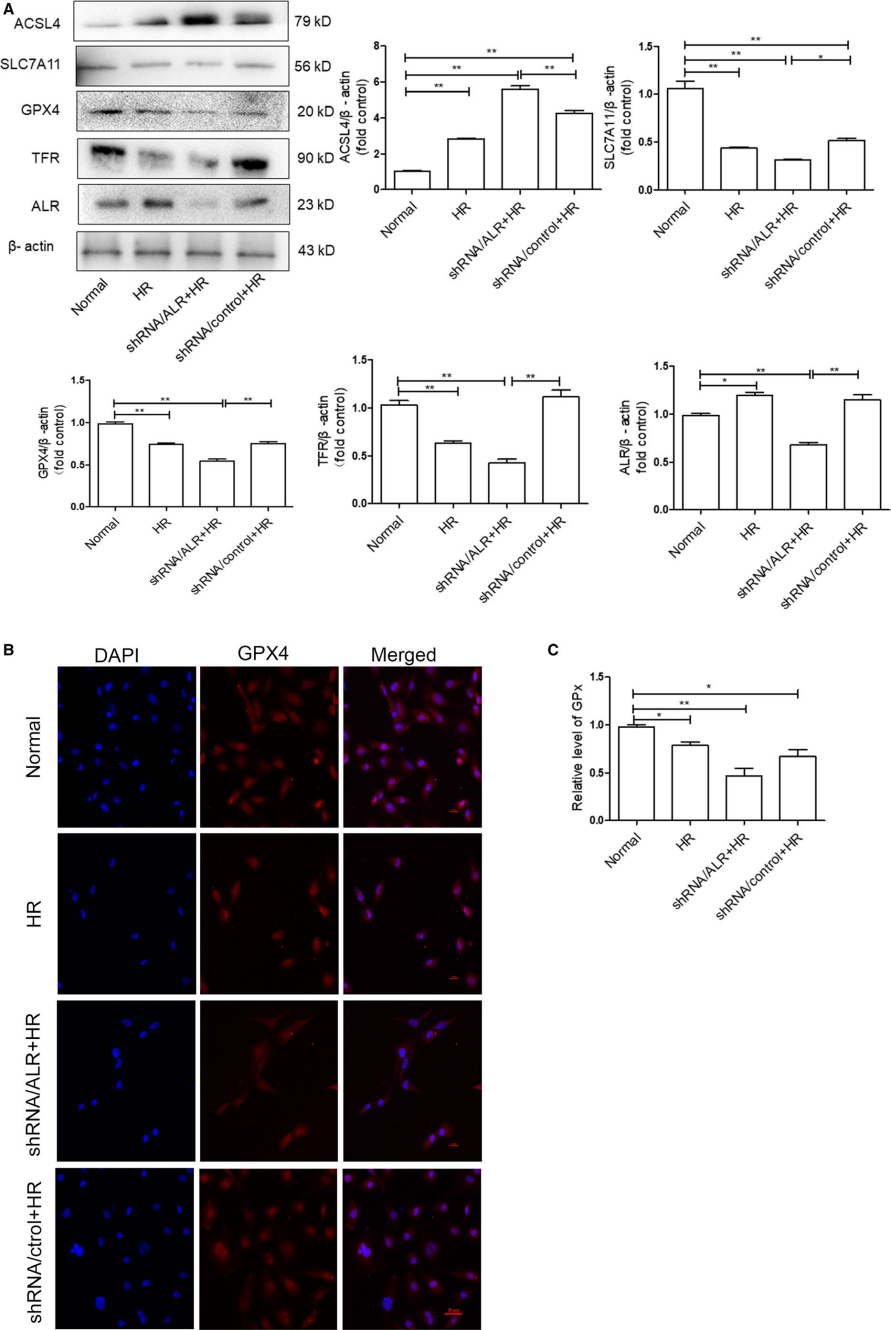
Silencing of augmenter of liver regeneration (ALR) aggravates I/R induced ferroptosis in vitro. A, The expression of acyl‐CoA synthetase long‐chain family member 4 (ACSL4), SLC7A11, glutathione peroxidase‐4 (GPX_4_) and transferrin receptor (TFR) was analysed by Western blotting in human kidney proximal tubular cells. (**P < 0.01 and *P < 0.05). B, GPX_4_ expression and localization after silencing ALR and I/R injury. GPX_4_ expression was examined by confocal laser‐scanning microscopy, scale bar = 20 μm. C, Relative content of GPXs. Data are presented as the mean ± SD of three independent experiments. (*P < 0.05 and **P < 0.01)

The expression of ferroptosis‐related proteins was more notable at reperfusion for 12 hours (R12) than at other time‐points; therefore, H6R12 was selected as the time‐point to observe ferroptosis after silencing ALR in HK‐2 cells. To further elucidate the protective role of ALR in ferroptosis, quantification of ACSL4, SLC7A11, GPX_4_ and TFR protein expression, which are ferroptosis‐related proteins, was performed by Western blotting after incubation for H6R12. As expected, the results showed significant differences in the expression of ACSL4, SLC7A11, GPX_4_ and TFR between the shRNA/ALR and shRNA/control groups (Figure 5A). Additionally, the expression of GPX_4_ was assumed to be similar for both the immunoblot analysis and immunofluorescence, which showed that the expression of GPX_4_ also gradually decreased after exposure to I/R and significantly decreased in the shRNA/ALR and shRNA/control groups. Furthermore, GPX_4_ was mainly localized to the nucleus and distributed diffusely throughout the cytoplasm based on confocal laser‐scanning microscopy (Figure 5B). These results suggested that ferroptosis was more active after I/R treatment in vitro. Additionally, silencing ALR up‐regulated ferroptosis in HK‐2 cells, and the activity of GPXs was significantly decreased compared with those of the shRNA/control groups (Figure 5C).

**Figure 6:**
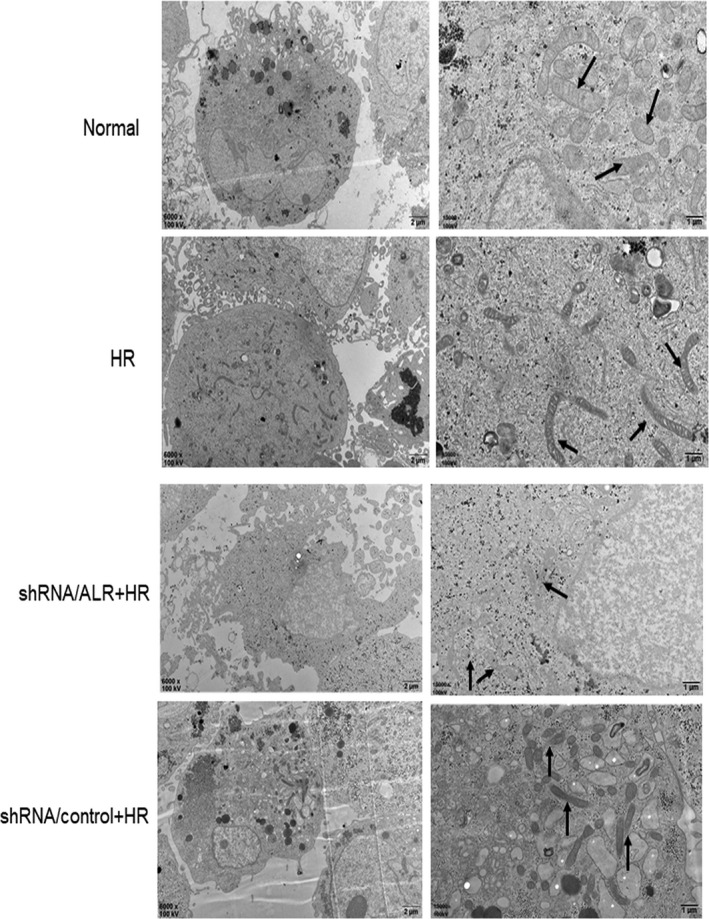
Transmission electron microscopy images in the short hairpin RNA lentivirals (shRNA)/augmenter of liver regeneration (ALR) groups and shRNA/control groups subjected to H/R. Magnification is × 6,000 or × 15,000, scale bars = 2 μm or 1 μm, respectively

Ferroptosis is a new form of regulatory cell death, with unique mitochondrial changes considered to be the main morphologic feature distinguishing it from other cell death modes. After HK‐2 cells were subjected to I/R, ferroptosis was characterized by the appearance of smaller than normal mitochondrial and darker‐staining membranes, with disorganization and even a reduction of mitochondrial crista. Notably, after silencing ALR, clear changes were observed in the morphology of mitochondria of the shRNA/ALR groups compared with that of the shRNA/control groups (Figure 6).

### 3.6 Inhibition of system xc‐ and silencing of ALR aggravate ferroptosis

**Figure 7:**
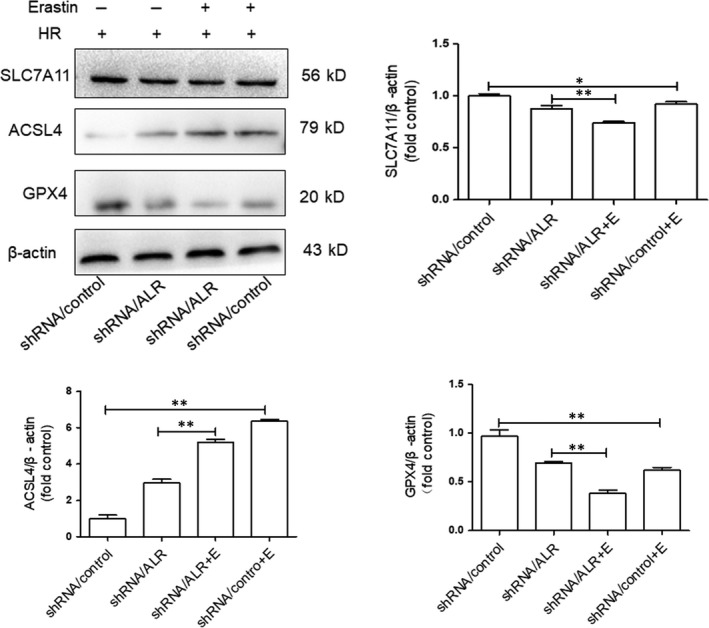
Ferroptosis inhibition and silencing of augmenter of liver regeneration (ALR) enhanced ferroptosis in human kidney proximal tubular cells at H6R12. SLC7A11, acyl‐CoA synthetase long‐chain family member 4 (ACSL4) and glutathione peroxidase‐4 (GPX_4_) expression was determined by immunoblotting. Data are shown as the mean ± SD.**P < 0.01 and *P < 0.05

Based on our previous findings, it is suggested that ALR plays a protective role in ferroptosis. To further clarify the potential protective mechanism of ALR in ferroptosis, erastin, a specific ferroptosis inducer, was used to inhibit system xc‐. After HK‐2 cells were treated with erastin (5 μmol/L) and knock down of ALR was performed, the change in ferroptosis was significant. There was also evidence of SLC7A11, ACSL4 and GPX_4_ protein expression. The expression of SLC7A11 and GPX_4_ was attenuated and that of ACSL4 was increased, particularly in the shRNA/ALR group, after exposure to erastin at H6R12 compared with those in the shRNA/control groups (Figure 7).

### 3.7 Mapping ALR in HK‐2 cells and ALR interaction with GPX_4_

**Figure 8:**
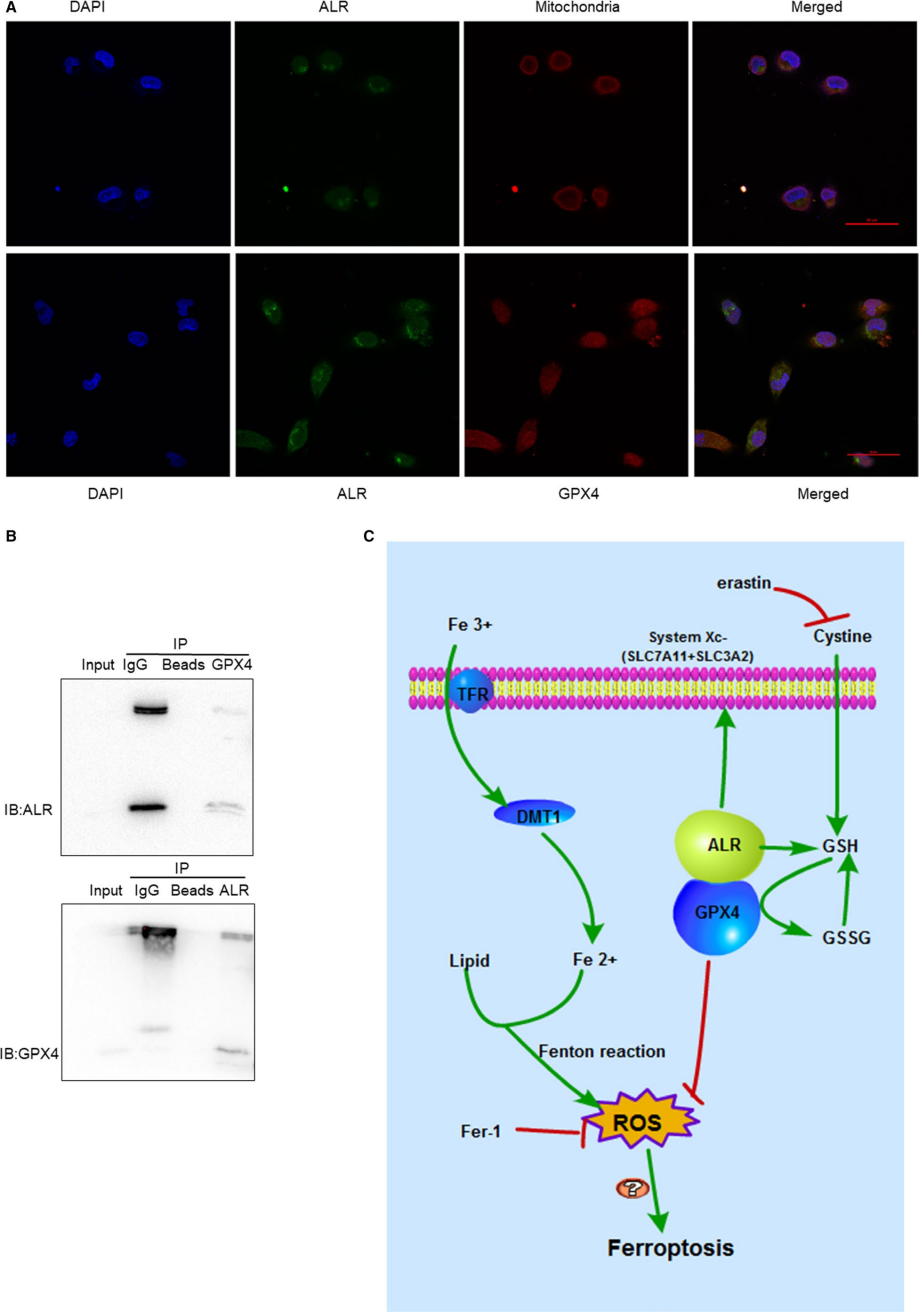
Augmenter of liver regeneration (ALR) interacts with glutathione peroxidase‐4 (GPX_4_) and the signalling pathway for ferroptosis. A, Mapping ALR and colocalization of ALR and GPX_4_ using confocal laser‐scanning microscopy. ALR is located in the mitochondria. ALR and GPX_4_ colocalization in the cytoplasm, the bar scale represents 50 μmol/L. B, Endogenous GPX_4_ and endogenous ALR in intact human kidney proximal tubular cells by co‐immunoprecipitation approach. C, ALR mediates the signalling pathways of ferroptosis

In our previous study, ALR was highly relevant to ferroptosis and silencing ALR enhanced ferroptosis in HK‐2 cells after I/R treatment in vitro. Moreover, GPX_4_ plays an essential role in ferroptosis. Further study is warranted to elucidate the interplay between GPX_4_ and ALR. Compelling evidence indicates that ALR resides in the intermembrane space of mitochondria as a sulfhydryl oxidase enzyme.^32^, ^33^ Our results are consistent with previous findings. We also observed ALR localization in mitochondria using confocal laser‐scanning microscopy (Figure 8A). Furthermore, colocalization of ALR and GPX_4_ was also observed in HK‐2 cells (Figure 8A). As an additional step to test the interaction of ALR with GPX_4_, the physical association between endogenous ALR and endogenous GPX_4_ was examined using a co‐immunoprecipitation approach (Co‐IP). (Figure 8B).

## 4 DISCUSSION

Ferroptosis is a novel form of RCD that involves disordered iron‐dependent accumulation of lipid peroxides, executed by polyunsaturated fatty acids (PUFA).^34^, ^35^ Finally, elevated ROS levels disrupt metabolism and primary physiological functions and can cause cell death and tissue impairment. ROS is a general term for oxygen atoms or clusters, including superoxide radical, hydroxyl radical (HO) and the non‐radical species hydrogen peroxide (H_2_O_2_).^36^ Compelling evidence has demonstrated that ferroptosis is involved in various conditions leading to AKI, that is, ischaemia‐reperfusion (I/R),^18^ rhabdomyolysis‐induced AKI^11^ and folic acid‐induced AKI.^12^ However, there are other forms of RCD involved in AKI, such as autophagy, apoptosis and necroptosis.^13^, ^35^ Some modulators, such as P53, participate in both apoptosis and ferroptosis in cancer cells.^37^, ^38^ Additionally, autophagy promotes ferroptosis via the degradation of ferritin in fibroblasts and cancer cells.^39^ Many ferroptosis regulators, such as GPX_4_, ACSL4, SC7A11 and Nrf1, have also been reported to potentially function in the regulation of autophagy.^40^ Elucidating the relationships between the various forms of RCD may provide new insights to therapeutic strategies to treat RCD associated diseases.

Ferroptosis, as a novel form of RCD, has its own unique morphologic, biochemical and genetic hallmarks. There are two main signalling pathways in ferroptosis^21^ (Figure 8). One is iron metabolism, and the other is the GPX_4_ system. Circulating iron, ferric iron (Fe^3+^), is imported into cells by the TFR, and then, Fe^3+^ is converted to ferrous iron (Fe^2+^) in the endosome. Excessive amounts of Fe^2+^ trigger ferroptosis by Fenton reactions with the accumulation of ROS. By contrast, GPX_4_ and system xc‐ may play a crucial role in ferroptosis. System xc‐ belongs to the family of heterodimeric amino acid transporters and is distributed in the renal tubules.^41^ The transporter is composed of two subunits, xCT (SLC7A11) and SLC3A2 (4F2hc); however, the light chain, SLC7A11, functions to exchange L ‐cystine and L ‐glutamate.^20^, ^42^ Erastin, a small molecule ferroptosis inducer, inhibits the function of system xc‐ and depletes GSH, finally inactivating GPX_4._ 16, 17, 18, 21, 43 In addition, GPX_4_ is a member of the GPXs family that inhibits lipid peroxidation and catalyses the transformation of GSH to GSSH, protecting cells from oxidative damage.^44^, ^45^, ^46^, ^47^ As an enzyme, the function of GPX4 depends on the system xc‐ as known as glu/cys antiporter in the plasma membrane.^48^ Mechanistically, how lipid peroxidation elicits ferroptosis requires further study. In the present study, there is increasing evidence that I/R injury involves ferroptosis. The expression of SLC7A11, TFR and GPX_4_ significantly decreased and that of ACSL4 significantly increased compared with those of the normal group (Figure 1). Concomitantly, the levels of ROS increased over time, and Fer‐1, a specific ferroptosis inhibitor, decreased lipid ROS to some extent (Figure 2B). Importantly, after I/R treatment, clear morphological changes in mitochondria were observed and accompanied by the appearance of smaller than normal mitochondria, darker‐staining membranes and disorganization with a reduction of mitochondrial crista (Figure 6).

ALR, as a cytokine, is a member of the ALR/Erv1 protein family with sulfhydryl oxidase activity.^49^ ALR has two isoforms, 15 KD and 23 KD, and the 23 KD ALR is mainly located in the membrane gap of mitochondria,^32^ consistent with our immunofluorescence results (Figure 8). An early study reported that ALR/ERV1 was involved in GSH and iron regulation.^33^, ^50^, ^51^ In our study, similar results were observed and showed that the GPX_S_ content and intracellular ROS levels were significantly increased after silencing ALR in HK‐2 cells. Moreover, ALR functions in anti‐oxidative stress reactions, consistent with our previous study in H_2_O_2_ induced oxidative models.^52^ Additionally, after silencing the ALR gene, mitochondria injury was aggravated (Figure 6) and the biomarker ACSL4 was significantly increased (Figure 5A). In summary, these findings suggested that ALR could scavenge ROS and played a critical role in ferroptosis in I/R induced AKI. To further understand the protective mechanisms of ALR in AKI, knock down of ALR and inhibition of system xc‐ with erastin (5 μm) promoted HK‐2 cell ferroptosis (Figure 7A). Furthermore, ALR and GPX_4_ colocalized in the cytoplasm (Figure 8A), and a strong signal was observed in Co‐IP (Figure 8B). Therefore, it is possible that ALR has protective effects in vitro via the GSH‐GPX_4_ system. In conclusion, the present study, for the first time, showed that I/R‐induced kidney ferroptosis was mediated by ALR via the GSH‐GPX4 system, which may shed light on the prevention and therapy of I/R induced AKI.

## 5 CONCLUSIONS

With this research work, we show for the first time that silencing ALR aggravates ferroptosis in an in vitro model of I/R. Notably, we show that I/R induced kidney ferroptosis is mediated by ALR, which is linked to the glutathione‐glutathione peroxidase (GSH‐GPx) system.

## AUTHORS’ CONTRIBUTIONS

LLH performed the experiments and wrote the manuscript. HS performed, measured and analysed the confocal laser‐scanning microscopy experiments. XHL revised and corrected the manuscript. XJ helped in writing the manuscript. LZ and QL designed the study, helped in analysing and interpretation of data. All authors read and approved the manuscript. Figure 2A and Figure 2B, XJ generated the flow cytometer and quantitated data. Figure 5B and Figure 8A, HS generated the confocal laser‐scanning microscopy data and labelled the image. Additionally, LLH generated the rest of the figures. LZ, the corresponding author, behalf of all authors approved the manuscript and all authors agree on the order in the manuscript.

## CONFLICT OF INTEREST

The authors have no conflicts of interest or financial interests to declare.
